# Zinc Nanocomposite Supported Chitosan for Nitrite Sensing and Hydrogen Evolution Applications

**DOI:** 10.3390/polym15102357

**Published:** 2023-05-18

**Authors:** Nada S. Al-Kadhi, Mahmoud A. Hefnawy, Sherif S. Nafee, Fowzia S. Alamro, Rami Adel Pashameah, Hoda A. Ahmed, Shymaa S. Medany

**Affiliations:** 1 Department of Chemistry, College of Science, Princess Nourah bint Abdulrahman University, P.O. Box 84428, Riyadh 11671, Saudi Arabia; 2 Chemistry Department, Faculty of Science, Cairo University, Giza 12613, Egypt; 3 Physics Department, Faculty of Science, King Abdulaziz University, Jeddah 21589, Saudi Arabia; 4 Department of Chemistry, Faculty of Applied Science, Umm Al-Qura University, Makkah 24230, Saudi Arabia

**Keywords:** chitosan composite, nitrite electrochemical sensor, hydrogen production, zinc oxide nanoparticle, Zn-chitosan composite

## Abstract

Nanoparticles of ZnO-Chitosan (Zn-Chit) composite were prepared using precipitation methods. Several analytical techniques, such as scanning electron microscope (SEM), transmitted electron microscope (TEM), powder X-ray diffraction (XRD), infrared spectroscopy (IR), and thermal analysis, were used to characterize the prepared composite. The activity of the modified composite was investigated for nitrite sensing and hydrogen production applications using various electrochemical techniques. A comparative study was performed for pristine ZnO and ZnO loaded on chitosan. The modified Zn-Chit has a linear range of detection 1–150 µM and a limit of detection (LOD) = 0.402 µM (response time ~3 s). The activity of the modified electrode was investigated in a real sample (milk). Furthermore, the anti-interference capability of the surface was utilized in the presence of several inorganic salts and organic additives. Additionally, Zn-Chit composite was employed as an efficient catalyst for hydrogen production in an acidic medium. Thus, the electrode showed long-term stability toward fuel production and enhanced energy security. The electrode reached a current density of 50 mA cm^−2^ at an overpotential equal to −0.31 and −0.2 V (vs. RHE) for GC/ZnO and GC/Zn-Chit, respectively. Electrode durability was studied for long-time constant potential chronoamperometry for 5 h. The electrodes lost 8% and 9% of the initial current for GC/ZnO and GC/Zn-Chit, respectively.

## 1. Introduction

Nitrite is a crucial element for food additives, concrete admixtures, and the cycling of soil [1,2]. The World Health Organization (WHO) has classified nitrite as a highly hazardous substance because of its potential to threaten human health (such as through carcinogenesis) and cause water and soil contamination over time [3]. Furthermore, nitroaromatic hydrocarbons, nitramines, and nitrates, which comprise the most used high-energy explosives, are mostly degraded by nitrite, which is one of the key components of improvised explosives [4]. The urgent requirement for on-site detection of trace nitrite is driven by the need to ensure sustainable environmental development and safeguard public health and security [5,6,7,8]. Many attempts have been undertaken to measure nitrite below one micromolar, such as by high-performance liquid chromatography, surface-enhanced Raman spectroscopy, fluorescence spectroscopy, and electrochemical detection [9,10].

Although most of these techniques perform well in lab settings, electrochemical methods are very sensitive and selective for detecting drugs and contaminated water [11,12,13,14]. Various benefits include affordability, reaction speed, simplicity, and reproducibility [15,16]. Using nanomaterials has led to the development of different electrochemical systems, including those incorporating nanostructured metal oxides [17]. Zinc oxide (ZnO) is a substance easily used in medical and biological applications, such as in wearable technology. ZnO is one of the greatest promising semiconductor materials for electrochemical sensor fabrications because of its high capacity to react with oxygen [18]. Several Zn-based electrodes were employed for the electrochemical detection of nitrite, such as flower-ZnO [19], Zn-Schiff base [20], ZnS [20], and ZnTiO_3_ [21]. 

Chitosan, a derivative of chitin, is commonly utilized in different applications. Recent developments in fermentation technology have enabled the production of novel chitosan with physiochemical properties distinct from waste materials, thereby offering a viable alternative to conventional sources such as crab shells. Chitosan is commonly used for immobilization due to its favorable environmental characteristics, high absorption capacity, remarkable layer-forming capabilities, superior permeability, heightened thermal stability, robust mechanical strength, biocompatibility, and convenient accessibility [22]. Chitosan exhibits distinctive structural and functional characteristics, such as non-toxicity, hydrophilicity, superior adhesion, biocompatibility, environmental sustainability, antibacterial and antimicrobial properties, and non-carcinogenicity, which render it highly versatile and applicable across diverse domains. This substance holds considerable importance in various fields, such as biomedicine, sensor technology, cosmetics, biochemistry, biotechnology, pharmaceuticals, food additives, preservative materials, water purification, antiseptic dyes, and agricultural applications [23,24,25,26,27].

Hydrogen gas (H_2_) plays a crucial role in facilitating the shift towards a sustainable energy system, owing to its ability to serve as a high-energy density for renewable energy [28,29,30]. Although water electrolysis presents an attractive option for green hydrogen production utilizing renewable energy sources, its global contribution to hydrogen production remains limited. This is primarily due to the requirement for costly electrocatalysts to offset the Ohmic losses associated with the kinetic overpotential of the system [31,32,33]. The literature reports zinc-based electrodes as an efficient surface for hydrogen production, such as ZnMn_2_O_4_, Zn-Ni-P, and Zn-AgIn_5_S_8_ [34,35,36]. 

In the present work, a composite of Zn-Chitosan is prepared as a dual functional catalyst for electrochemical detection of nitrite and hydrogen production enhancement. The prepared catalyst was characterized using several electrochemical techniques. Different kinetics parameters are estimated to evaluate the process efficiency. Additionally, real nitrite samples were used to construct calibration curves. A comparative study was performed for pristine ZnO and Zn-Chitosan composites for hydrogen production in an acidic medium.

## 2. Materials and Methods

### 2.1. Preparation of ZnO Nanoparticles

Using a hydrothermal process, hexagonal nanocubes of zinc oxide were created. In a typical experiment, 1.2 g of Zn(NO_3_)_2_·6H_2_O were dissolved in double-distilled water, followed by the slow addition of 0.1 M of KOH. This resulted in a mixture of 100 mL, which was then agitated for 20 min while being dispersed in an ultrasonic water bath for 30 min. The mixture was then placed into a Teflon-lined stainless-steel autoclave, which was then dried in an oven before undergoing a 12-h hydrothermal treatment at 180 °C. The item was filtered, cleaned with ethanol and water, and then dried in an oven at 60 °C.

### 2.2. Preparation of Zn-Chit Composite

Zn-Chit composite was prepared by a mixture of chitosan solution mixed with ZnO nanoparticles: 0.5 g of chitosan (Sigma Aldrich, St. Louis, MO, USA, 50,000–190,000 Da (based on viscosity), and 75–85% deacetylated) was added to 40 mL absolute ethanol in a 250 mL beaker, and then the temperature was raised gently with stirring. 0.5 g of ZnO nanoparticles were added to the mixture. The solution temperature was cooled to room temperature. The addition of the chitosan to the chitosan solution led to crosslinking and the encapsulation of ZnO nanoparticles. Chitosan in acidic media becomes a polyelectrolyte because of the protonation of the –NH_2_ groups. The following equilibrium reaction describes the state of ionization. Therefore, about 1 mL of acetic acid (10%) was added to the mixture and stirred until the solution turned viscous. After ten minutes, the mixture underwent filtration and was rinsed with distilled water. The product was dried in an oven set at 80 °C for 3 h.

### 2.3. Fabrication of Electrode

A glassy carbon electrode with a diameter of 3 mm (0.0707 cm^2^) was used as the working electrode. It was first polished with gentle emery paper and cleaned with ethanol and double-distilled water. After that, the cast solution was created by dispersing 20 mg of the catalyst powder (ZnO or Zn-Chit) in 0.5 mL of ethanol and 0.5 mL of 5 wt% Nafion using an ultrasonic bath for 1 h. The modified electrode was made in the following manner: GC/ZnO and GC/Zn-Chit are accomplished by a drop cast of 20 µL of catalyst solution onto electrode’s surface, allowing it to dry for 4 h at 50 °C. The Autolab PGSTAT128N was used to perform all electrochemical experiments. The electrochemistry program NOVA (Version 2.1, Metrohm Autolab, Utrecht, The Netherlands) fits the impedance spectrum. Ag/AgCl/KCl (sat.) and Pt wire were employed as reference and counter electrodes.

GC/ZnO and GC/Zn-Chit were also used as working electrodes. All potential values in this work were compared to a reference electrode made of Ag/AgCl/sat.KCl for nitrite electrochemical sensor application. Furthermore, the potential was referenced to a reversible hydrogen electrode (RHE) for hydrogen evolution reaction (HER) applications. The electrochemical impedance spectroscopy measurements were adjusted to a constant AC voltage value using an AC voltage amplitude of 10 mV and a frequency range of 1 × 10^4^ Hz to 0.1 Hz.

The potential was referenced to the reversible hydrogen electrode (RHE) regarding the following equations [37]:

E_RHE_ = E_Ag/AgCl_ + E° _Ag/AgCl_ + 0.059 pH (1)

Electrochemical experiments were carried out in a 0.5 M supporting electrolyte solution with H_2_SO_4_. The potential was standardized to a hydrogen electrode that is reversible as follows:

E_RHE_ = E_Ag/AgCl_ + 0.197       (0.5 M H_2_SO_4_ ~ pH = 0) & (E° _Ag/AgCl_ = 0.197 V) (2)

## 3. Result & Discussion

### 3.1. Catalyst Characterization

**Figure 1:**
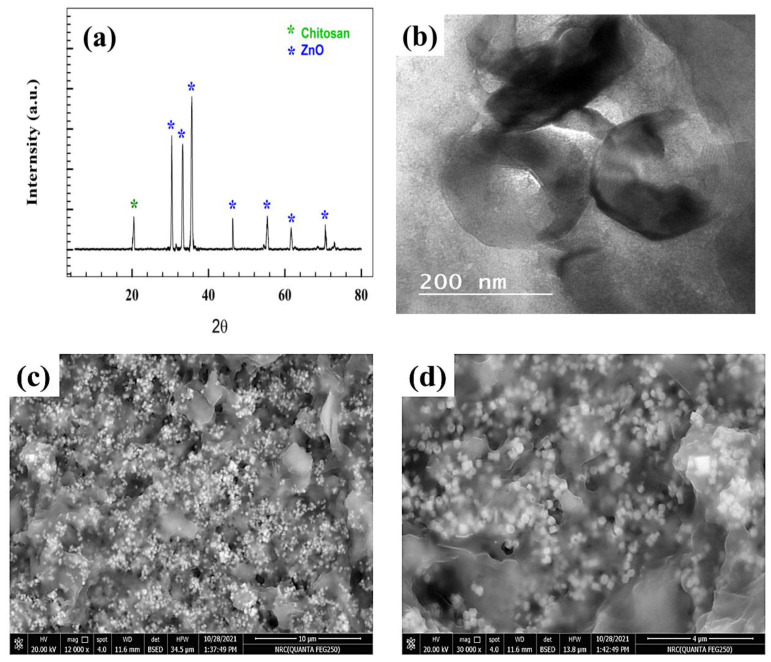

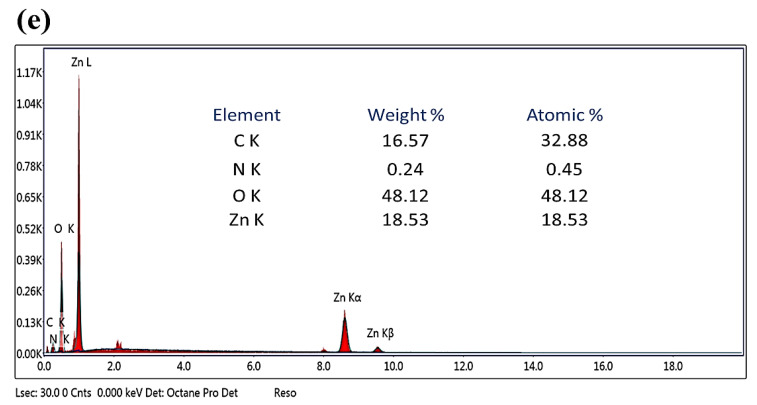
(**a**) Zn-Chit XRD, (**b**) TEM, (**c**,**d**) SEM, (**e**) EDX.

The structure of modified Zn-Chit materials was determined using X-ray diffraction, as illustrated in Figure 1. The study reports the observation of seven maxima for ZnO, with respective Miller indices of {100}, {002}, {101}, {102}, {110}, {103}, and {112}, at 2θ = 31, 34, 36, 47, 56, 62, and 69, as documented in reference [38]. Furthermore, the observed peak at a 2θ value of approximately 20 degrees can be attributed to the crystallographic plane with Miller indices of {110} for chitosan, as reported in previous studies [39]. Moreover, the homogeneous and consistent dispersion of ZnO onto chitosan substrate promotes the adsorption of nitrite onto the electrode interface. Transmission electron microscopy (TEM) was employed as the conventional technique for determining the dimensions of ZnO nanoparticles. The mean particle size of zinc oxide was estimated to be around 70 nm. (Figure 1b). 

The scanning electron microscope (SEM) was utilized to characterize the surface morphology of Zn-Chit, as illustrated in Figure 1c,d. The scanning electron microscopy (SEM) images of ZnO nanoparticles incorporated within chitosan sheets revealed the presence of hexagonal nanocubes. 

Energy-dispersive X-ray spectroscopy (EDX), a technique for elemental analysis, was employed to identify the existence of elements, including Zn, C, N, and O (see Figure 1e). The atomic ratios of Zn and O suggest the existence of ZnO and the lack of adulteration from other constituents in the specimens. Additionally, the presence of the carbon and nitrogen on surface attributed to the chemical structure of chitosan.

**Figure 2:**
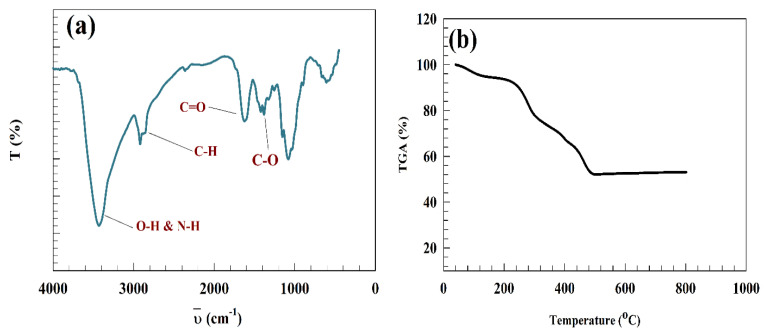
Representation of (**a**) IR spectra of Zn-Chit. (**b**) TGA curve of Zn-Chit.

Zn-Chit samples were characterized using FT-IR, revealing a range of absorption bands that facilitate identifying specific functional groups. Figure 2a illustrates the infrared spectra of the Zn-Chit sample. The stretching vibrations observed at wave number 3460 cm^−1^ were attributed to O–H and N–H bond-stretching while the stretching of C–H was identified at 2941 cm^−1^. The absorption bands observed at 1633, 1573, 1436, and 1374 cm^−1^ correspond to the C=O stretching of the amide band, accompanied by the bending of N–H, C–H, and O–H, respectively. The spectral analysis revealed that the band at 1162 cm^−1^ corresponded to the anti-symmetric stretching of the (C–O–C) bridge.

Additionally, the skeletal vibrations involving C–O stretching were predicted to occur at 1084 and 1023 cm^−1^ [40,41]. The spectrum of synthesized ZnO nanoparticles reveals a fundamental vibration mode at 3425 cm^−1^, identified as O–H stretching and deformation. This mode is associated with the adsorption of water on the metal surface. The tetrahedral coordination of Zn is accountable for its absorption at 873 cm^−1^. The vibrational stretching of ZnO nanoparticles is indicated by the observed bands within the range of 732 to 609 cm^−1^.

A thermal analytical technique, namely TGA, was utilized to estimate the thermal durability and chemical decomposition. As depicted in Figure 2b, the thermogravimetric analysis curve of Zn-Chit is presented. The composite material exhibited four distinct thermal transitions at 82, 277.2, 388.4, and 454.2 °C. The thermal transition observed at approximately 85 °C is commonly attributed to the water removal. Additionally, it has been reported that the thermal decomposition of chitosan occurs through a two-stage process [42]. It is anticipated that the degradation of chitosan will result in thermal decomposition peaks at 277.2 and 388.4 °C. The fourth thermal transition within the temperature range of 454.2 °C is associated with ZnO. 

### 3.2. Zn-Chit Composite for Nitrite Detection

**Figure 3:**
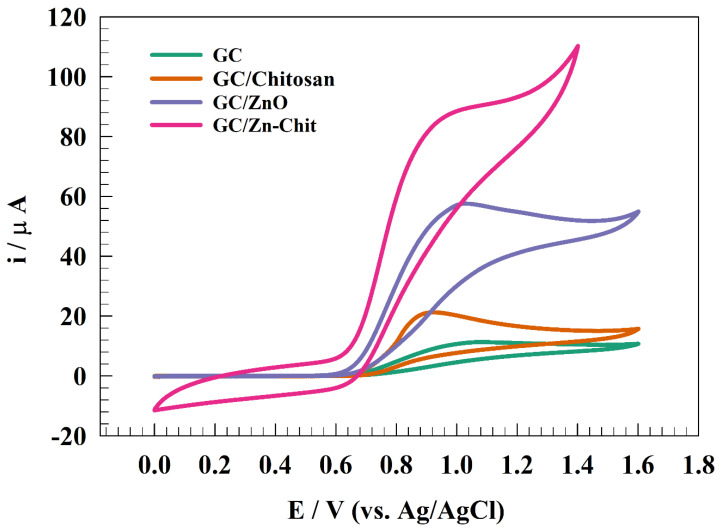
Comparison between different surfaces for nitrite electrochemical detection.

The present study investigated the efficacy of various modified GC electrodes, specifically GC/Chitosan, GC/ZnO, and GC/Zn-Chit, in detecting nitrites through the cyclic voltammetry (CV) technique. The experiment was utilized in a solution of 0.05 M phosphate buffer (pH = 7) in the presence of 50 µM of nitrite, with a sweep rate of 10 mV s^−1^. One oxidation peak was observed in the potential range 0.8~1.0 V (vs. Ag/AgCl). For instance, the estimated Eps were observed to be 1.01, 0.94, 1.02, and 0.99 V for GC, GC/Chitosan, GC/ZnO, and GC/Zn-Chit, respectively. The forward peak indicates the transformation of N$O_{2}^{-}$ into N$O_{3}^{-}$. The electrochemical detection of nitrite is deemed irreversible due to an undefined backward peak and the lack of reduction peaks. Figure 3 shows the activity of the Zn-Chit surface toward nitrite detection compared to pristine ZnO counterparts. However, the well distribution of ZnO on chitosan sheets enhanced the detection process by increasing the compatibility between the ZnO and the glassy carbon electrode.

Furthermore, chitosan facilitates the adsorption of ions from bulk to surface. However, several chitosan composites were reported in the literature as efficient surfaces for nitrite detection in an aqueous medium, such as graphene/polypyrrole/chitosan, Au-chitosan, and Au@RGO-Chitosan [43,44,45]. Therefore, adding the chitosan to ZnO nanoparticles enhanced the anodic oxidation current of the nitrite from 58 to 92 µA. Moreover, the activity of bare glassy carbon and chitosan was studied for nitrite detection. The adsorption step is essential for the electrochemical process [46]. Furthermore, chitosan showed lower activity toward nitrite detection than ZnO due to the absence of active centers for the charge transfer process. 

**Figure 4:**
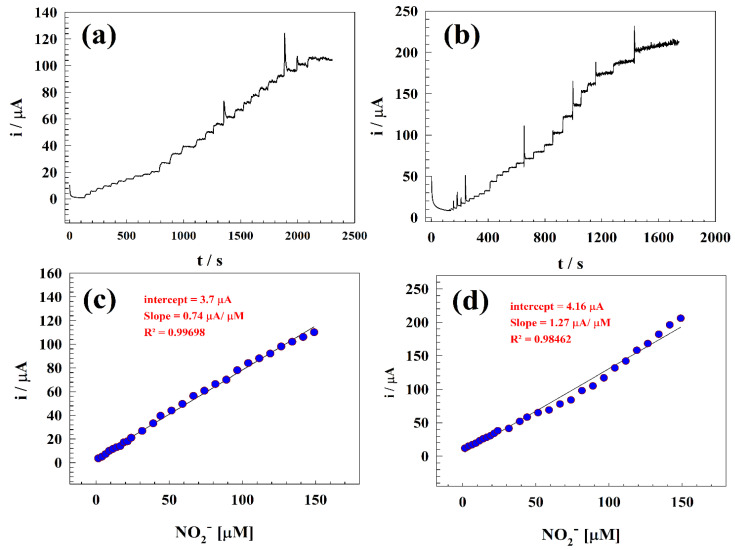
Chronoamperograms of (**a**) GC/ZnO and (**b**) GC/Zn-Chit. Calibration curves of (**c**) GC/ZnO and (**d**) GC/Zn-Chit in concentration ranges of 1 to 150 µM.

Since the electrode surface’s sensitivity toward nitrite is considered as an essential parameter for the study, the calibration curve was studied for different surfaces in the presence of least amount of nitrite. The limit of detection is a method of analysis that can calculate the smallest amount of analyte detected in a sample [47]. Contrarily, the limit of quantization is the lowest drug concentration that can be quantitatively detected with the claimed accuracy and precision [48]. Various values (from 1 µM to 150 µM) were used to produce the calibration curve for nitrite ion detection and sensitivity determination. The chronoamperometric method was used to determine the limit of detection and linear dynamic range by gradually introducing nitrite into drinking water. The electrochemical behavior of the GC/ZnO- and GC/Zn-Chitosan-modified electrodes was investigated by observing their constant potential chronoamperograms. The experiments were conducted in PBS with a pH of 7 and a concentration of 0.05 M at a constant potential of 0.9 V (vs. Ag/AgCl), as illustrated in Figure 4a,b. Figure 4c,d illustrates the relationship between the nitrite concentrations and oxidation current within a concentration range of 1–150 µM nitrite. Furthermore, the slope of the calibration curve was used to assess the limits of quantization and detection.

Equations (3) and (4) represent the linear ranges for the modified electrodes GC/ZnO and GC/Zn-Chit, as follows:

i_(µA)_ = 0.74 C_ZnO_ (µM) + 3.7 (3)

i_(µA)_ = 1.27 C_Zn-Chit_ (µM) + 4.16 (4)

Additionally, using the equations LOD = 3 s/m and LOQ = 10 s/m, respectively, the detection and quantization limits are computed [15], where m is the slope of the calibration curve and s is the standard deviation. The LOD and LOQ for electrodes were determined as 0.689 µM and 2.3 µM for GC/ZnO, 0.402 µM, and 1.34 µM for GC/Zn-Chit, respectively.

The modified GC/ZnO and GC/Zn-Chit electrodes’ linear dynamic range and limit of detection for determining nitrite were compared to those of other results reported in the published literature, and the results are shown in Table 1.

**Figure 5:**
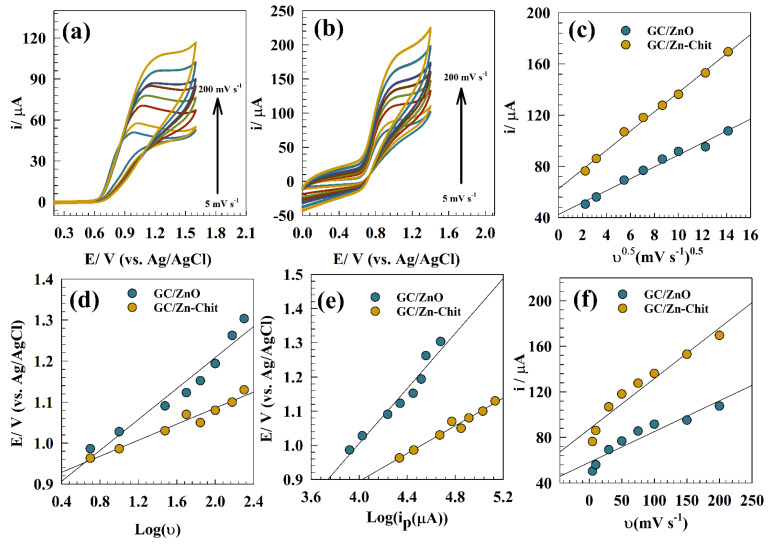
CV of (**a**) GC/ZnO and (**b**) GC/Zn-Chit at different scan rates (5 to 200 mV s^−1^). (**c**) Linear relation between the square root of scan rate and nitrite current. (**d**) Linear relation between logarithm of scan rate and anodic peak potential, (**e**) linear relation between Log (ip) vs. peak potential. (**f**) Linear relation between scan rate vs. peak current of nitrite.

For a complete understanding of nitrite sensing, kinetic parameters were estimated for nitrite oxidation over the modified electrodes. Figure 5a,b show Cvs. of the modified GC/ZnO and GC/Zn-Chit in a solution of 0.05 M PBS and 50 µM of nitrite at a wide range of scan rate (0.005 to 0.2 V s^−1^ (vs. Ag/AgCl)). 

The diffusion coefficient (D) can be estimated using the Randles–Sevcik equation for irreversible process as follows [56,57,58,59,60,61,62]:

I_p_ = 2.99 × 10^5^ n A C_o_ [(1 − α) n_o_ D ʋ] ^0.5^ (5)

where, i is the nitrite’s oxidation current, n is the number of electrons (n = 1), A is the electrode’s surface area, D is the analyte diffusion coefficient, C_o_ is the analyte concentration, and ν is the scan rate.

The diffusion coefficient was estimated using Randles–Sevick by constructing the linear relation between the nitrite oxidation current versus the square root of the scan rate (see Figure 5c). The provided diffusion coefficients are 6.3 × 1$0^{-5}$ and 2.4 × 1$0^{-4}$ cm^2^ s^−1^ for GC/ZnO and GC/Zn-Chit electrodes, respectively. A higher diffusion coefficient value for a chitosan-based surface regards the chitosan’s higher ability to adsorb the nitrite. 

Figure 5d represents a linear relation between peak potential versus the logarithm of scan rate for different modified surfaces. Thus, reversibility can be confirmed by the shift of the Ep positively as the scan rate increases [63]. The shift in the peak potential’s position was observed by increasing the scan rate values according to the Laviron equation for irreversible reactions [63,64,65,66,67]:


(6) $$E_{\mathrm{pa}}(V)\;=\;E{}^{\circ}\;-\;\frac{RT}{\alpha nF}\;\ln\;\frac{RTk_{s}}{\alpha nF}\;+\;\frac{RT}{\alpha nF}\;\ln\;\nu$$

E_pa_ is the peak potential, R is the universal gas constant, E° is the formal potential, T is the temperature, n is the number of electrons, v is the scanning rate, and F is the Faraday constant.

The transfer coefficient (α) is the kinetic parameter that infers the reaction’s propensity for moving in the oxidation/reduction direction. When (α) is smaller than 0.5, the direction of oxidation is preferred. Considering the Laviron relation, the transfer coefficients were calculated using the linear relationship between Log (v) and Epa as 0.32 and 0.61 for modified GC/ZnO and GC/Zn-Chit, respectively. However, the symmetry factor, charge transfer coefficient (α), indicates that the oxidation of nitrite upon GC/Zn-Chit is more favorable compared to its GC/ZnO counterparts. The interaction between electrode and nitrite was established by linear relation between E_p_ vs. Log(i), and I_p_ vs. scan rate. (see Figure 5e,f). Whereas, the linear relation reflects the adsorption of nitrite on electrode surfaces [68,69]. Additionally, reaction is considered to be controlled by mixed adsorption/desorption process. 

**Figure 6:**
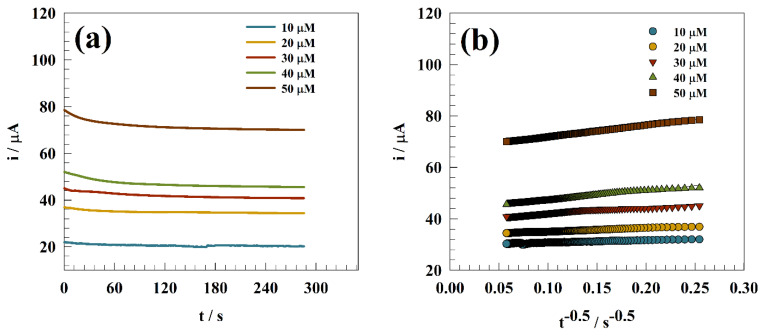
(**a**) Chronoamperograms of GC/Zn-Chit for different nitrite concentrations. (**b**) Linear relation between t^−0.5^ and nitrite current.

Figure 6a shows the chronoamperograms of GC/Zn-Chit for different nitrite concentrations at constant potential of 900 mV (vs. Ag/AgCl). however, the constant potential chronoamperometery technique was employed to estimate the diffusion coefficients for nitrite toward the modified electrode.

The Cottrell equation [70,71,72] was used to determine the average diffusion coefficient of nitrite molecules at a GC/Zn-Chit-modified electrode at a potential of 0.85 V (vs. Ag/AgCl) in a solution of 0.05 M PBS and several nitrite concentrations, as follows:

i = nFAC_o_D^0.5^(π) ^−0.5^ (t)^−0.5^ (7)

where, i is the current, n is the number of oxidizing electrons (n = 1), F is the Faraday constant and A is the electrode surface area, and t is the operating time. As represteneted in Figure 6b, diffusion coefficient estimated using the slope of the Cottrell relation. Whereas, the provided diffusion values for nitrite equaled 6.1 × 10^−4^, 3.07 × 10^−4^, 4.7 × 10^−4^, 6.2 × 10^−4^, and 5.4 × 10^−4^ cm^2^ mol^−1^ for 10, 20, 30, 40, and 50 µM, respectively. 

**Figure 7:**
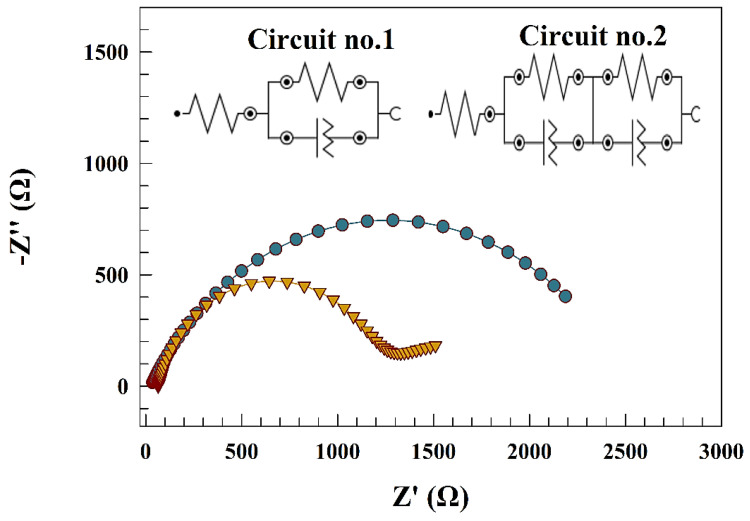
Nyquist plot of different surfaces, inset figure: fitting circuits.

The activity of both electrodes was utilized by EIS. Figure 7 represents Nyquist plot for the GC/ZnO and GC/Zn-Chit surfaces at a constant potential of 0.85 V (vs. Ag/AgCl). However, EIS data was fitted using NOVA software. The inset figure represents the fitting circuits of the corresponding Nyquist results. However, circuit number one is attributed to the GC/ZnO electrode that included solution resistance (Rs) connected in series with a cell of charge transfer resistance (Rc) connected to a constant phase element (CPE). On the other hand, Nyquist data for GC/Zn-Chit fitted by circuit number two includes solution resistance (Rs) connected to two cells of resistance and a constant phase element. Whereas the presence of two cells is attributed to two layers of surface behavior. However, the Nyquist plot’s diameter reflects the electrode’s activity. The lower diameter represents the higher charge transfer process and respectable activity toward nitrite sensing. The charge transfer resistances of 2480.8 and 1192.3 Ω were evaluated for GC/ZnO and GC/Zn-Chit electrodes, respectively. Table 2 contains the reported fitting data for the modified electrodes.

**Figure 8:**
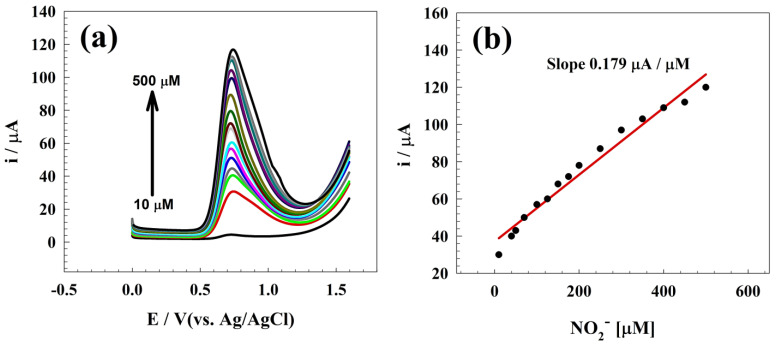
(**a**) DPV of nitrite detection in milk sample for modified surface GC/Zn-Chit. (**b**) Calibration curve of nitrite in the milk sample.

Real samples were employed to determine the electrodes’ activity in real-time applications. Thus, three types of samples were prepared for electrochemical analysis in milk. In the pre-treatment process of milk, a mixture of 15 g milk and 2 mL semi-saturated ammonium sulfate solution was subjected to a temperature of 65 °C for 15 min to induce protein precipitation. Subsequently, the acquired supernatant was diluted to a volume of 45 mL using a 0.1 M K_2_S_2_O_8_/PBS (pH 6.0) solution and administered [73]. Figure 8a shows the DPV of the different spiked nitrite concentrations in milk samples at a wide range of nitrite concentrations (10 to 500 µM). The calibration curve was established and attributed to the milk sample, as illustrated in Figure 8b. Linear dynamic regions were detected at 10–500 µM with corresponding limit of detections equal to 8.82 µM. In order to perform electrochemical detection for the real samples, the standard addition technique was employed. This involved the addition of various concentrations of nitrite (20, 80, 150, 230, and 300 μM) to the treated extracts from milk. Table 3 shows the real sample detection recovery. 

**Figure 9:**
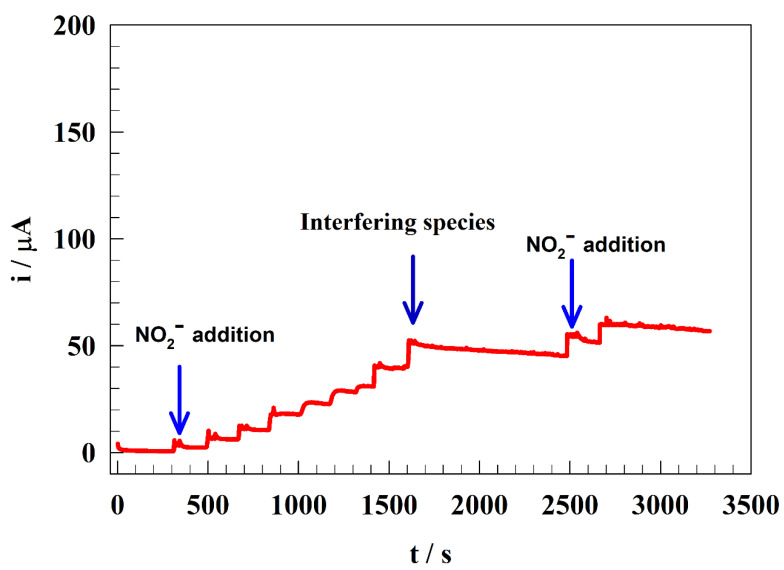
Chroamperogram for GC/Zn-Chit after and before adding interfering species.

To examine the anti-interference capabilities of the sensor, a GC/Zn-Chit sensor was utilized to measure a variety of potentially interfering substances, including inorganic ions such as CuSO_4_, MgCl_2_, CaCl_2_, NaCl, and NH_4_Cl, as well as organic substances such as ascorbic acid, uric acid, glucose, and dopamine. Figure 9 depicts the chronoamperogram of the modified electrode GC/Zn-Chit. Firstly, nitrite solution was spiked to the solution, whereas the electrode response was observed as an increase in current. Then, the interfering solutions were added to the solution with only a slight change in the measured current. Finally, the reactivity of the electrode was investigated by spiking different concentrations of nitrite in the solution. Thus, the observed current increased again, reflecting the electrode surface’s regeneration and the electrode’s high anti-interference ability. 

### 3.3. Zn-Chit Composite for Hydrogen Production

**Figure 10:**
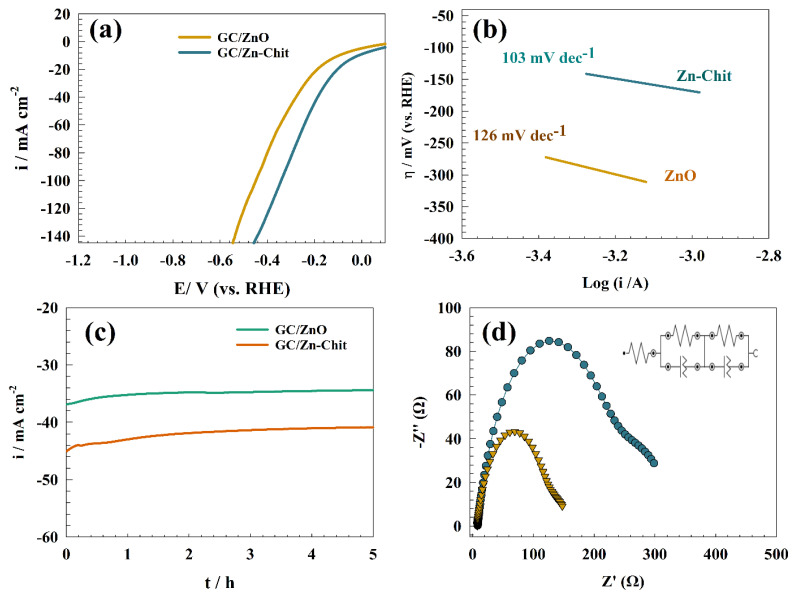
(**a**) LSV of GC/ZnO and GC/Zn-Chit in an acidic medium for hydrogen evolution, (**b**) Tafel slopes of HER, (**c**) Long-term chronoamperometry of modified GC/ZnO and GC/Zn-Chit, (**d**) Nyquist plot of GC/ZnO and GC/Zn-Chit electrodes for HER and corresponding fitting circuit.

Hydrogen evolution reactions on GC/ZnO- and GC/Zn-Chit-modified surfaces were investigated. Figure 10a represents the LSV of the modified electrodes in a solution of 0.5 M H_2_SO_4_ at a scan rate of 10 mV s^−1^. However, the presence of chitosan and ZnO composite enhanced the efficiency of the hydrogen production process. Whereas, the mechanism of hydrogen evolution mainly consists of adsorption steps, as follows [74,75]:


(8) $$\mathrm{Volmer}\;\mathrm{step}:\;H^{+}+e^{-}\to H_{ads}$$

(9) $$\mathrm{Tafel}\;\mathrm{step}:\;2H_{ads}\to H_{2}$$

(10) $$\mathrm{Heyrovsky}\;\mathrm{step}:\;H^{+}+H_{ads}+e^{-}\to H_{2}$$

Regarding the previously mentioned equations, the first stage of the HER process requires the adsorption of hydrogen ions (Volmer step) on the electrode surface. Next, two hydrogen ions that have been adsorbed on the surface are either recombined (Tafel step) or a direct bond is formed between an adsorbed hydrogen atom and a hydrated proton in the medium (Heyrovsky step).

Accordingly, chitosan was reported to enhance the catalyst’s ability to generate more hydrogen. For instance, a combination between metal oxides was found to shift the overpotential of hydrogen production toward a more positive value that indicates the reaction became more thermodynamically favored, such as CdS-Chitosan, CoNPs-Chitosan, and Pt-Chitosan-TiO_2_ [76,77,78]. By comparing pristine and chitosan-modified electrodes, both electrodes reached a current density of 50 mA cm^−2^ at over potential of −0.31 and −0.2 V (vs. RHE) for GC/ZnO and GC/Zn-Chit, respectively. On the other hand, a Tafel slopes curve was established for different surfaces to determine each electrode’s thermodynamic favorability (see Figure 10b). Consequently, the Tafel slopes provided for GC/ZnO and GC/Zn-Chit electrodes are 103 and 126 mV dec^−1^, respectively. Figure 10c shows the long-term stability of HER upon modified GC/ZnO and GC/Zn-Chit electrodes for 5 h. The chronoamperometry was performed in a solution of 0.5 M H_2_SO_4_ at a potential of −0.3 V (vs. RHE). High electrode stability was observed, and the outcome current density decreased by 8% and 9% for GC/ZnO and GC/Zn-Chit electrodes, respectively. Electrochemical impedance spectroscopy (EIS) was employed to characterize HER upon modified GC/ZnO and GC/Zn-Chit electrodes. As represented in Figure 10d, Nyquist plot of modified GC/ZnO and GC/Zn-Chit electrodes in a solution of 0.5 M H_2_SO_4_ at constant potential equaled −0.3 V (vs. RHE). The EIS data were fitted by NOVA software. The inset of Figure 10d illustrates the fitting circuit for the modified electrode. The equivalent circuit for HER consisted of solution resistance (Rs) connected in parallel with two similar resistance circuits and a constant phase element (CPE). The presence of a constant phase element corresponds to the surface roughness of the electrode. The following are essentially identical parameters for the constant phase element and capacitance [79]:

(11) $$Z=\frac{1/Yo}{{\left(j\omega \right)}^{\alpha }}$$

where, constant phase element and capacitance are equaled (α = 1). However, the charge transfer resistances for GC/ZnO and GC/Zn-Chit are 216 and 110 Ω, respectively. The fitted EIS data is reported in Table 4 for GC/ZnO and GC/Zn-Chit. 

## 4. Conclusions

The chitosan-based composite was successfully prepared for nitrite sensing and hydrogen production applications. Comparative studies between pristine and chitosan-based ZnO showed a synergistic effect between chitosan and ZnO nanoparticles. The modified GC/Zn-Chit electrodes showed efficient activity toward nitrite detection with a linear detection range of 1 to 150 µM and a detection limit of 0.402 µM. Therefore, a synergistic effect between ZnO nanoparticles and chitosan membrane could be described by the outstanding activity of modified GC/Zn-Chit toward nitrite detection. The electrode showed anti-interference ability in the presence of several metal ions and organic additives. The ability of nitrite detection was studied for milk as real samples. Furthermore, the modified surfaces found efficient activity toward hydrogen production. The electrode reached 50 mA cm^−2^ at overpotentials of −0.31 and −0.2 V (vs. RHE) for GC/ZnO and GC/Zn-Chit, respectively.

## Figures and Tables

**Table 1:** Comparison of different surfaces for nitrite sensing.

Electrode	Linear Detection Range (µM)	Limit of Detection (µM)	Method	Reference
CeO_2_/La_2_O_3_	0.25 to 4000	0.015	Amperometry	[49]
Au-MoS_2_@RGO	0.2 to 2600	0.038	Amperometry	[50]
GC/PANI/NiOnF	1–500	0.064	Amperometry	[51]
MoO_3_-Co_3_O_4_	0.3125 to 4514	0.075	Amperometry	[52]
Cobalt-NFs	100 to 2150	1.2	Amperometry	[53]
SiO_2_-Fe_3_O_4_	0.72–110	0.74	Amperometry	[54]
PdO@RGO	10 to 1500	10.14	differential pulse voltammetry	[55]
GC/ZnO	1 to 150	0.689	Amperometry	This work
GC/Zn-Chitosan	1 to 150	0.402	Amperometry	This work

**Table 2:** EIS fitting parameters for ZnO and Zn-Chit electrodes for nitrite sensing.

Electrode	R_s_	R_1_	Q_1_		R_2_	Q_2_	
	Ohm	Ohm	Y_0_	N	Ohm	Y_0_	m
GC/ZnO	27.238	2480.8	0.00013008	0.68866	-	-	-
GC/Zn-Chitosan.	43.505	640	0.002161	0.43496	1192.3	0.0000921	0.86971

**Table 3:** Recovery results for the real samples.

Sample	Added (µM)	Found (µM)	Recovery (%)
Milk	20	19	95
	80	82	102
	150	146	97
	230	235	102
	300	297	99

**Table 4:** EIS fitting parameters for ZnO and Zn-Chit electrodes for HER.

Electrode	R_s_	R_c_	Q_1_		R_2_	Q_2_	
	Ohm	Ohm	Y_0_	N	Ohm	Y_0_	m
GC/ZnO	8.16	109.5	0.00046257	0.61097	216.49	0.0008012	0.79496
GC/ZnO-Chit	7.65	41.04	0.0010375	0.55337	110.9	0.0009921	0.80568

## Data Availability

The data used for research described in this manuscript are available upon request from corresponding authors: shymaasamir80@cu.edu.eg; shymaa@sci.cu.edu.eg (S.S.M). maadel@cu.edu.eg; maahefnawy@gmail.com (M.A.H).
